# Anesthetic Μanagement of an Emergency Craniotomy in a Patient With Noonan Syndrome: A Case Report

**DOI:** 10.7759/cureus.95601

**Published:** 2025-10-28

**Authors:** Maria Diakomi, Aikaterini Andreou, Alexandros Makris

**Affiliations:** 1 Department of Anesthesiology, General Hospital of Kavala, Kavala, GRC; 2 Department of Anesthesiology, Asklepieion Hospital of Voula, Voula, GRC

**Keywords:** awake intubation, case report, difficult airway, emergency craniotomy, noonan syndrome

## Abstract

A female patient with Noonan syndrome was evaluated for urgent surgical removal of an epidural hematoma, caused by a fall during a seizure attack. Preoperative evaluation revealed signs of a difficult airway, including abnormal craniofacial anatomy, short neck, and restricted neck mobility. In the operating room, endotracheal intubation with a McGrath MAC video-laryngoscope was attempted following the administration of 40 mcg dexmedetomidine (i.e., 1 mcg/kg infused in 10 minutes), 25 mcg fentanyl, and 1 mg midazolam. No desaturation or other adverse effects were recorded till the end of the procedure. This case report highlights critical anesthetic considerations of a patient with Noonan syndrome in the setting of a difficult airway, urgent surgical intervention, and limited patient cooperation. Airway manipulation of this surgical population necessitates careful preparation and planning. Anesthetic technique should be individualized according to the specific clinical manifestations of the syndrome.

## Introduction

Noonan syndrome (NS) is an autosomal dominant genetic disorder affecting 1 in 1000-2,500 child births, caused by mutations in genes encoding proteins of the RAS /MAPK signaling pathway, regulating cell growth and survival. Anesthetic management of patients with NS poses a significant challenge due to the wide range of organ system involvement, including craniofascial features associated with the possibility of a difficult airway, congenital heart defects affecting hemodynamic stability, developmental delay, skeletal issues, and bleeding disorders that could affect the administration of spinal anesthesia [1]. We report a patient with NS presenting for urgent surgical removal of an epidural hematoma. Our case report provides valuable knowledge for anesthesiologists to anticipate and manage patients with a rare condition like NS, especially in emergency settings.

## Case presentation

**Figure 1:**
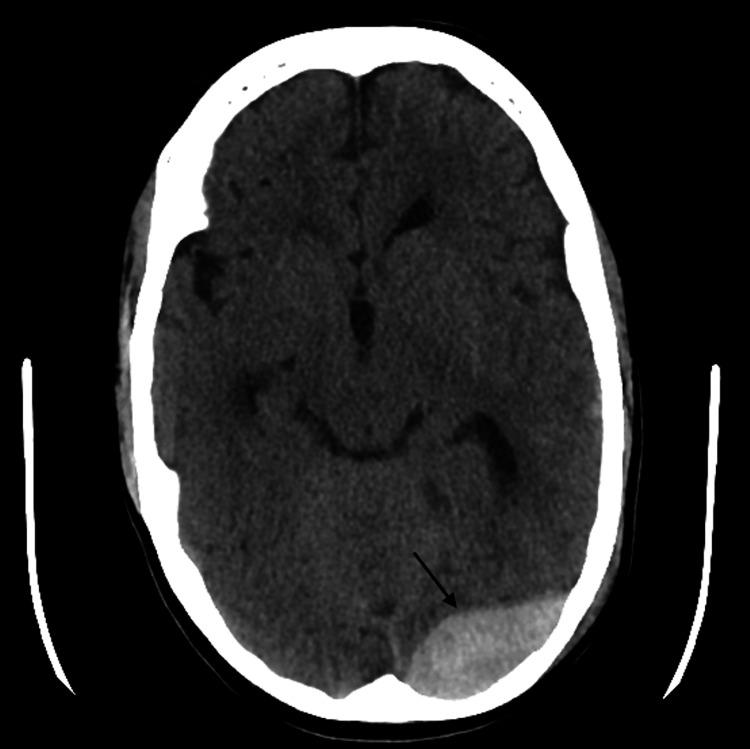
Preoperative axial CT scan showing the location of the epidural hematoma (black arrow), without significant midline deviation

A 21-year-old female with NS (40 kg, 155 cm) with congenital pulmonary valve stenosis treated with balloon valvuloplasty at the age of 6 months and ureterocele, was evaluated for urgent surgical removal of an epidural hematoma, caused by a fall during a seizure attack. On the CT scan, she had no signs of raised intracranial pressure and no significant midline deviation (Figure 1).

**Figure 2:**
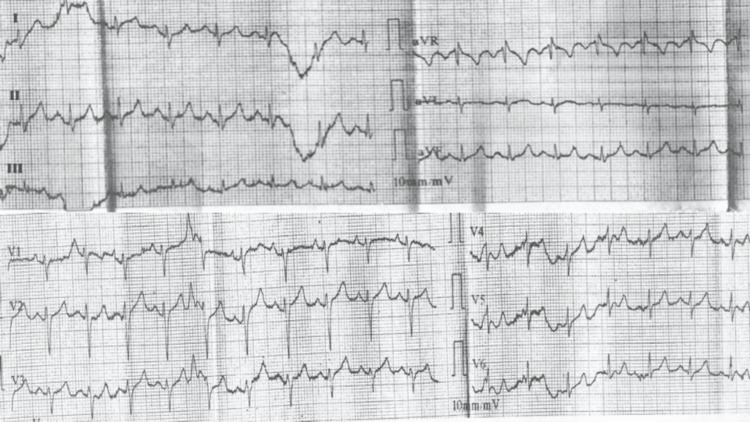
12-lead ECG showing normal sinus rhythm, without ischemic findings

Her medical history included intellectual disability and seizures that started five years ago, managed with levetiracetam 250 bid. Her electrocardiogram showed a normal sinus rhythm with no ischemic changes (Figure 2).

Laboratory test values were within normal limits. Her last meal intake took place more than six hours before the seizure activity.

In the preoperative evaluation, she had an abnormal craniofacial anatomy, including micrognathia, an arched hard palate, protruding central incisors, a short neck, and restricted neck mobility. Mouth opening was approximately a two-finger width. The cooperation of the patient was not good enough to evaluate the Mallampatti score. In light of these findings, a scrubbed ENT surgeon and a second anesthesiologist were called to be present during the induction of anesthesia.

In the operating room, after establishment of standard monitoring and venous access, 40 mcg dexmedetomidine (i.e., 1 mcg/kg infused in 10 minutes), 25 mcg fentanyl, and midazolam 1 mg in 0.5 increments were administered. In addition to standard monitoring, sedation level was monitored with the bispectral index (BIS). The trolley with all the equipment necessary for difficult airway management was ready.

At BIS 60-65, a successful attempt at endotracheal intubation (6.5 mm internal diameter tube) with preservation of spontaneous ventilation was carried out using a McGrath MAC video-laryngoscope (Aircraft Medical, UK, blade size 3), followed by the administration of 2 mg/kg propofol and 0.6 mg/kg rocuronium. No desaturation occurred during the procedure, which lasted 166 minutes. The patient's oxygen saturation levels were 99-100%, the systolic blood pressure was maintained at 110-115 mmHg, and the heart rate at 75-80 bpm.

Anesthesia was maintained with sevoflurane 1 MAC, 45% oxygen/air, and continuous infusion of remifentanil (0.02 mcg/kg/min) until the end of craniotomy, aiming at preserving adequate cerebral perfusion pressure, with mean arterial pressure above 70 mmHg and keeping BIS values within 40-60. At the end of the operation, the patient was transferred to the ICU, and the next morning, she was extubated without any complications. The rest of the postoperative period in the ward was uneventful. Neurological assessment was normal, and the patient was discharged on the fourth postoperative day.

## Discussion

Patients with NS are managed through a team-based approach, focusing on regular cardiac monitoring and treatment of heart defects, treatment of developmental issues with early intervention, supportive therapies, and possibly growth hormone replacement and screening for bleeding, endocrine, and hearing/vision problems, with ongoing genetic counseling and follow-up [1].

The anesthetic decision-making presented here provides a safe and successful strategy for anesthesiologists to follow in patients with NS on an urgent basis. However, there is a lack of generalizability because this is a case report. Despite this, it should be kept in mind that the scenario of anticipated difficult airway management, in urgent cases of uncooperative patients with NS, is uncommon in anesthesiologic literature [2,3]. Moreover, most of the published cases report successful bag mask ventilation following induction of anesthesia without a neuromuscular blocking agent, while our case reports the infusion of drugs aiming at maintaining spontaneous ventilation and preparing the patient for awake intubation.

NS belongs to a group of disorders caused by mutations in genes involved in the Ras/mitogen-activated protein kinase (Ras/MAPK) pathway, which regulates the cell cycle and cellular growth. The diagnosis and differential diagnosis of NS are essentially based on the revised van der Burgt clinical criteria [1]. Key features of the syndrome reflect the diverse downstream ramifications of this mutated signal pathway and include facial and cardiac abnormalities associated with a possible difficult airway and cardiac compromise, respectively [4].

The anesthetic management of these patients should include meticulous preoperative evaluation of anatomic abnormalities and pathophysiologic derangements. Our patient was spared abnormal cardiac findings and symptoms, probably due to the previous successful surgical management. However, the characteristic facial anatomy suggesting a possible difficult airway was further complicated by the urgency of surgery and aspiration risk.

Numerous other NS syndrome sequelae should be excluded, including skeletal, hematological, and neurological manifestations. Thoracic cage deformities may be present in more than 60% of cases, impairing not only spontaneous but also mechanical ventilation. Hematological manifestations that could lead to abnormal bleeding during surgery include thrombocytopenia and myeloproliferative diseases. Other clinical entities that could be encountered are ocular abnormalities, dermatological symptoms (hyperkeratosis, eczema), renal impairment, malignancies like leukemia and jaw tumors, hydrocephalus, Arnold-Chiari I malformation, and atlanto-axial dislocation. The coexistence of many of these abnormalities renders anesthetic management of NS patients quite challenging [5-7].

The anesthesiologist in our case proceeded with an awake intubation strategy according to expected difficult airway management guidelines, despite the patient’s low level of cooperation [8]. Equipment for cricothyroidotomy and tracheostomy was readily available, and specialized medical personnel were asked to participate during airway manipulation.

Intubation with video laryngoscopy was preferred to fiberoptic intubation, as video laryngoscopy provides a magnified, illuminated view of the glottis [9], making it particularly advantageous in such anatomically challenging cases. On the other hand, fiberoptic intubation requires a high level of patient cooperation for optimal visualization during awake intubation. In view of the patient’s intellectual disability and low level of cooperation, video laryngoscopy provided a more practical and time-efficient solution in the urgent setting. Additionally, the anesthesiologist’s experience and familiarity with the McGrath MAC video laryngoscope contributed to the decision, as operator expertise significantly influences the success rate and safety of airway management techniques [10,11]. This approach aligns with the American Society of Anesthesiologists (ASA) difficult airway guidelines, which recommend maintaining spontaneous ventilation in anticipated difficult airways [8].

## Conclusions

The anesthetic management of patients with NS requires an individualized anesthetic plan based on the specific clinical manifestations of the syndrome, including anatomic, cardiac, skeletal, hematological, and neurological derangements. Our case report focuses on critical considerations with respect to airway management of NS patients in the context of craniofacial abnormalities, urgent surgical intervention, and poor patient cooperation.

Taking into consideration the patient’s uneventful perioperative course, this case report provides an efficient and safe anesthetic plan, underscoring the importance of a pre-formulated airway strategy and a multidisciplinary approach in managing NS patients requiring urgent surgery. The awake intubation strategy in accordance with the anticipated difficult airway algorithm was challenged by the lack of cooperation. Additionally, the anesthesiologist’s familiarity and expertise contributed to the choice of video laryngoscopy over fiberoptic intubation. However, the presented decision-making cannot be generalized because this is the case of a single patient.
